# Form II of adipic acid–nicotinohydrazide (1/2)

**DOI:** 10.1107/S1600536811054043

**Published:** 2011-12-21

**Authors:** Andreas Lemmerer, Joel Bernstein, Volker Kahlenberg

**Affiliations:** a Molecular Sciences Institute, School of Chemistry, University of the Witwatersrand, Johannesburg, PO Wits 2050, South Africa; b Faculty of Science, NYU Abu Dhabi, PO Box 129188, Abu Dhabi, United Arab Emirates; c Institute of Mineralogy and Petrography, University of Innsbruck, Innsbruck 6020, Austria

## Abstract

The crystal structure of the title co-crystal, 2C_6_H_7_N_3_O·C_6_H_10_O_4_, is a second polymorph, designated form II, of the co-crystal formed between the two mol­ecules [Lemmerer *et al.* (2011 ▶). *CrystEngComm*, **13**, 55–59]. The asymmetric unit comprises one mol­ecule of nicotinic acid hydrazide, and one half-mol­ecule of adipic acid (the entire mol­ecule is completed by the application of a centre of inversion). In the crystal, mol­ecules assemble into a three-dimensional network of hydrogen bonds, formed by three N—H⋯O hydrogen bonds and one O—H⋯N hydrogen bond. The O—H⋯N hydrogen bond formed between the carboxyl group and the pyridine ring is supported by a C—H⋯O hydrogen bond.

## Related literature

For the first polymorph, see: Lemmerer *et al.* (2011 ▶). For experimental techniques, see: Friščić *et al.* (2009 ▶); Skovsgaard & Bond (2009 ▶); Karki *et al.* (2009 ▶). For hydrogen-bonding motifs, see: Bernstein *et al.* (1995 ▶).
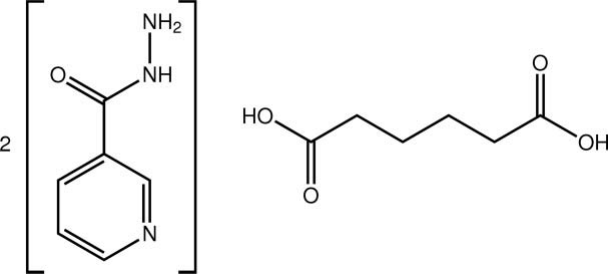


## Experimental

### 

#### Crystal data

2C_6_H_7_N_3_O·C_6_H_10_O_4_
*M* *_r_* = 420.43
Monoclinic, 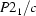

*a* = 15.9747 (4) Å
*b* = 7.3309 (2) Å
*c* = 8.7451 (2) Å
β = 103.729 (3)°
*V* = 994.87 (4) Å^3^
*Z* = 2
Mo *K*α radiation
μ = 0.11 mm^−1^
*T* = 173 K
0.32 × 0.28 × 0.04 mm

#### Data collection

Oxford Diffraction Xcalibur diffractometer with a Ruby (Gemini ultra Mo) detector
Absorption correction: multi-scan (*CrysAlis PRO*; Oxford Diffraction, 2006 ▶) *T*_min_ = 0.92, *T*_max_ = 0.98
6207 measured reflections
1845 independent reflections
1487 reflections with *I* > 2σ(*I*)
*R*_int_ = 0.020

#### Refinement

*R*[*F* ^2^ > 2σ(*F* ^2^)] = 0.029
*wR*(*F* ^2^) = 0.077
*S* = 1.02
1845 reflections
153 parameters
H atoms treated by a mixture of independent and constrained refinement
Δρ_max_ = 0.18 e Å^−3^
Δρ_min_ = −0.14 e Å^−3^

### 

Data collection: *CrysAlis PRO* (Oxford Diffraction, 2006 ▶); cell refinement: *CrysAlis PRO*; data reduction: *CrysAlis PRO*; program(s) used to solve structure: *SHELXS97* (Sheldrick, 2008 ▶); program(s) used to refine structure: *SHELXL97* (Sheldrick, 2008 ▶); molecular graphics: *ORTEP-3* (Farrugia, 1997 ▶) and *DIAMOND* (Brandenburg, 1999 ▶); software used to prepare material for publication: *WinGX* (Farrugia, 1999 ▶) and *PLATON* (Spek, 2009 ▶).

## Supplementary Material

Crystal structure: contains datablock(s) global, I. DOI: 10.1107/S1600536811054043/tk5035sup1.cif

Structure factors: contains datablock(s) I. DOI: 10.1107/S1600536811054043/tk5035Isup2.hkl

Supplementary material file. DOI: 10.1107/S1600536811054043/tk5035Isup3.mol

Supplementary material file. DOI: 10.1107/S1600536811054043/tk5035Isup4.cml

Additional supplementary materials:  crystallographic information; 3D view; checkCIF report

## Figures and Tables

**Table 1:** Hydrogen-bond geometry (Å, °)

*D*—H⋯*A*	*D*—H	H⋯*A*	*D*⋯*A*	*D*—H⋯*A*
N1—H1⋯O3^i^	0.877 (14)	2.174 (15)	3.0409 (14)	169.7 (11)
N3—H3*A*⋯O1^ii^	0.934 (14)	2.129 (14)	3.0349 (14)	163.0 (12)
N3—H3*B*⋯O1^iii^	0.888 (15)	2.258 (16)	3.1426 (14)	173.7 (13)
O2—H2⋯N2	0.950 (19)	1.671 (19)	2.6126 (13)	170.3 (16)
C2—H2*A*⋯O3	0.95	2.73	3.3838 (14)	126

Symmetry codes: (i) 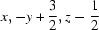; (ii) 
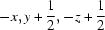
; (iii) 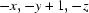.

**Fig. 1.**
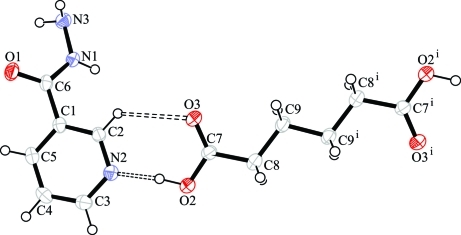
The asymmetric unit of (I) extended to show the entire dicarboxylic acid and showing the atomic numbering scheme. Displacement ellipsoids are shown at the 50% probability level. Symmetry code: (i): -x, -y, -z.

**Fig. 2.**
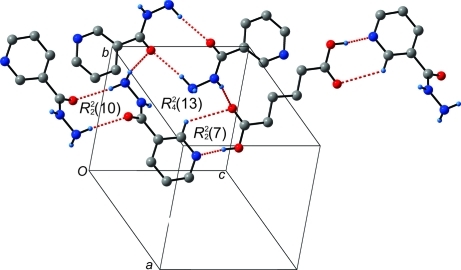
Hydrogen bonding diagram of (I), Form II, showing the various ring shaped hydrogen bonding motifs. Intermolecular N—H···N, O—H···N and C—H···O hydrogen bonds are shown as dashed red lines.

## supplementary crystallographic information

### Comment

Form I of the title compound was obtained by solution crystallization, where 1 equivalent of adipic acid was dissolved with two equivalents of niazid in methanol, and then the solution left to slowly evaporate over a few days (Lemmerer *et al.*, 2011). The ratio of the two starting materials was determined by the two expected primary hydrogen bond interactions, namely the two carboxylic acids hydrogen bonding to the single pyridine of two niazid molecules. Form II was obtained by first grinding the two starting materials in the same stoichiometric ratio as form I for 20 minutes, followed by conventional solution crystallization by dissolving the ground powder in methanol. Crystals were obtained after a few days from the methanol solution. Obtaining polymorphs by either grinding or solution crystallization is a focus of recent research (Friščić *et al.*, 2009; Skovsgaard & Bond, 2009; Karki *et al.*, 2009).

The crystallographic asymmetric unit of the resulting crystal structure of form II reflects this stoichiometry, containing one niazid molecule in a general position and one half adipic acid molecule on a special position (Fig. 1). The two molecules lie approximately co-planar. The expected heterosynthon is formed between two niazid and one adipic acid molecule lying on a crystallographic center of symmetry (which requires the pyridine ring and carboxylic acid to be co-planar). The heterosynthon between the dicarboxylic acid molecule and the pyridine ring is formed by a O—H···N hydrogen bond, as well as a C—H···O hydrogen bond, to form a *R*^2^_2_(7) ring (Bernstein *et al.*, 1995). The niazid molecules are connected by a centrosymmetric *R*^2^_2_(10) ring using N—H···O hydrogen bonds from one of the amine H atoms. Adjacent dimers are joined by a N—H···O bond using the second amine H atom, which together with the C—H···O hydrogen bond and the N—H···O hydrogen bond from the amide H forms a *R*^2^_4_(13) ring (Fig. 2). Hence, all four H bond donors are used to form a 3-D network. In form I, the same *R*^2^_2_(7) ring is observed but the amide H atom forms a *C*(4) chain to form a 2-D sheet, which is then connected into a 3-D network by the amine H atoms hydrogen bonding to adjacent sheets (Lemmerer *et al.*, 2011).

### Experimental

A stoichiometric amount in the ratio of 2:1 of nicotinic acid hydrazide to adipic acid was ground together in a mortar with a pestle under the drop-wise addition of methanol over 20 minutes. The resulting powder was then dissolved in 10 ml of AR-grade methanol, and the solution slowly left to evaporate to afford colourless block-like crystals after one week.

### Refinement

The C-bound H atoms were geometrically placed (C—H bond lengths of 0.95 (aromatic CH) and 0.99 (methylene CH_2_) Å) and refined as riding with *U*_iso_(H) = 1.2*U*_eq_(C). The N-bound and O-bound H atoms were located in the difference map and coordinates refined freely together with their isotropic displacement parameters.

### Crystal data

**Table:**

2C_6_H_7_N_3_O·C_6_H_10_O_4_	*F*(000) = 444
*M**_r_* = 420.43	*D*_x_ = 1.403 Mg m^−^^3^
Monoclinic, *P*2_1_/*c*	Mo *K*α radiation, λ = 0.71073 Å
Hall symbol: -P 2ybc	Cell parameters from 3572 reflections
*a* = 15.9747 (4) Å	θ = 3.1–28.5°
*b* = 7.3309 (2) Å	µ = 0.11 mm^−^^1^
*c* = 8.7451 (2) Å	*T* = 173 K
β = 103.729 (3)°	Block, colourless
*V* = 994.87 (4) Å^3^	0.32 × 0.28 × 0.04 mm
*Z* = 2	

### Data collection

**Table:**

Oxford Diffraction Xcalibur diffractometer with a Ruby (Gemini ultra Mo) detector	1487 reflections with *I* > 2σ(*I*)
ω scans	*R*_int_ = 0.020
Absorption correction: multi-scan (*CrysAlis PRO*; Oxford Diffraction, 2006)	θ_max_ = 25.5°, θ_min_ = 3.1°
*T*_min_ = 0.92, *T*_max_ = 0.98	*h* = −13→19
6207 measured reflections	*k* = −8→8
1845 independent reflections	*l* = −10→9

### Refinement

**Table:**

Refinement on *F*^2^	H atoms treated by a mixture of independent and constrained refinement
Least-squares matrix: full	*w* = 1/[σ^2^(*F*_o_^2^) + (0.048*P*)^2^] where *P* = (*F*_o_^2^ + 2*F*_c_^2^)/3
*R*[*F*^2^ > 2σ(*F*^2^)] = 0.029	(Δ/σ)_max_ < 0.001
*wR*(*F*^2^) = 0.077	Δρ_max_ = 0.18 e Å^−^^3^
*S* = 1.02	Δρ_min_ = −0.14 e Å^−^^3^
1845 reflections	Extinction correction: *SHELXL97* (Sheldrick, 2008), Fc^*^=kFc[1+0.001xFc^2^λ^3^/sin(2θ)]^-1/4^
153 parameters	Extinction coefficient: 0.0095 (18)
0 restraints	

### Special details

**Table:**

Experimental. Absorption corrections were made using the program Crysalis Empirical absorption correction using spherical harmonics, implemented in SCALE3 ABSPACK scaling algorithm in CrysAlisPro.
Geometry. All e.s.d.'s (except the e.s.d. in the dihedral angle between two l.s. planes) are estimated using the full covariance matrix. The cell e.s.d.'s are taken into account individually in the estimation of e.s.d.'s in distances, angles and torsion angles; correlations between e.s.d.'s in cell parameters are only used when they are defined by crystal symmetry. An approximate (isotropic) treatment of cell e.s.d.'s is used for estimating e.s.d.'s involving l.s. planes.

### Fractional atomic coordinates and isotropic or equivalent isotropic displacement parameters (Å^2^)

**Table:**

	*x*	*y*	*z*	*U*_iso_*/*U*_eq_	
C1	0.16016 (7)	0.37907 (15)	0.35826 (13)	0.0237 (3)	
C2	0.22411 (7)	0.44813 (15)	0.47997 (13)	0.0248 (3)	
H2A	0.2294	0.5767	0.4922	0.03*	
C3	0.27134 (8)	0.16066 (15)	0.56223 (15)	0.0296 (3)	
H3	0.3105	0.0841	0.6326	0.036*	
C4	0.20920 (8)	0.08110 (15)	0.44526 (15)	0.0317 (3)	
H4	0.2056	−0.0479	0.4355	0.038*	
C5	0.15231 (8)	0.19064 (16)	0.34265 (14)	0.0291 (3)	
H5	0.1082	0.1384	0.2622	0.035*	
C6	0.09817 (8)	0.49832 (15)	0.24735 (13)	0.0243 (3)	
N1	0.13026 (7)	0.65323 (12)	0.20370 (11)	0.0259 (2)	
H1	0.1859 (9)	0.6738 (17)	0.2300 (15)	0.030 (3)*	
N2	0.27881 (6)	0.34156 (13)	0.58100 (11)	0.0276 (3)	
N3	0.07997 (7)	0.77311 (15)	0.09121 (13)	0.0303 (3)	
H3A	0.0389 (9)	0.8250 (17)	0.1382 (16)	0.036 (4)*	
H3B	0.0508 (9)	0.7022 (19)	0.0145 (18)	0.042 (4)*	
O1	0.02225 (5)	0.45309 (11)	0.19861 (10)	0.0327 (2)	
C7	0.38377 (7)	0.67377 (15)	0.82289 (13)	0.0259 (3)	
C8	0.45756 (8)	0.75709 (16)	0.94010 (16)	0.0352 (3)	
H8A	0.5119	0.7085	0.9206	0.042*	
H8B	0.4543	0.7172	1.0467	0.042*	
C9	0.46178 (7)	0.96301 (15)	0.93861 (14)	0.0281 (3)	
H9A	0.4079	1.0133	0.9588	0.034*	
H9B	0.466	1.0045	0.833	0.034*	
O2	0.39428 (6)	0.49783 (11)	0.80455 (11)	0.0341 (2)	
O3	0.32068 (5)	0.75783 (10)	0.75195 (11)	0.0353 (2)	
H2	0.3478 (12)	0.449 (2)	0.727 (2)	0.069 (5)*	

### Atomic displacement parameters (Å^2^)

**Table:**

	*U*^11^	*U*^22^	*U*^33^	*U*^12^	*U*^13^	*U*^23^
C1	0.0214 (6)	0.0264 (6)	0.0251 (6)	−0.0031 (5)	0.0090 (5)	−0.0026 (5)
C2	0.0240 (6)	0.0226 (6)	0.0279 (7)	−0.0018 (5)	0.0062 (5)	−0.0018 (5)
C3	0.0293 (7)	0.0270 (7)	0.0342 (7)	0.0028 (5)	0.0105 (6)	0.0043 (5)
C4	0.0374 (8)	0.0216 (6)	0.0387 (7)	−0.0023 (5)	0.0144 (6)	−0.0018 (5)
C5	0.0280 (7)	0.0281 (6)	0.0317 (7)	−0.0065 (5)	0.0083 (5)	−0.0057 (5)
C6	0.0221 (7)	0.0279 (6)	0.0231 (6)	−0.0031 (5)	0.0055 (5)	−0.0062 (4)
N1	0.0207 (6)	0.0276 (6)	0.0280 (6)	−0.0013 (4)	0.0030 (5)	0.0001 (4)
N2	0.0245 (6)	0.0274 (6)	0.0301 (6)	−0.0012 (4)	0.0048 (4)	0.0009 (4)
N3	0.0284 (6)	0.0319 (6)	0.0290 (6)	0.0001 (5)	0.0038 (5)	0.0023 (5)
O1	0.0232 (5)	0.0372 (5)	0.0346 (5)	−0.0062 (4)	0.0010 (4)	0.0019 (4)
C7	0.0228 (7)	0.0276 (7)	0.0266 (6)	0.0012 (5)	0.0042 (5)	0.0027 (5)
C8	0.0291 (7)	0.0348 (8)	0.0350 (7)	0.0021 (5)	−0.0058 (6)	−0.0030 (5)
C9	0.0206 (7)	0.0321 (7)	0.0292 (7)	0.0011 (5)	0.0014 (5)	−0.0023 (5)
O2	0.0296 (5)	0.0276 (5)	0.0382 (6)	0.0027 (4)	−0.0059 (4)	−0.0015 (4)
O3	0.0244 (5)	0.0306 (5)	0.0444 (5)	0.0030 (4)	−0.0047 (4)	0.0000 (4)

### Geometric parameters (Å, °)

**Table:**

C1—C2	1.3845 (16)	N1—H1	0.877 (14)
C1—C5	1.3908 (15)	N3—H3A	0.934 (14)
C1—C6	1.4932 (16)	N3—H3B	0.888 (15)
C2—N2	1.3371 (15)	C7—O3	1.2180 (13)
C2—H2A	0.95	C7—O2	1.3154 (13)
C3—N2	1.3382 (15)	C7—C8	1.4965 (17)
C3—C4	1.3747 (18)	C8—C9	1.5113 (16)
C3—H3	0.95	C8—H8A	0.99
C4—C5	1.3744 (17)	C8—H8B	0.99
C4—H4	0.95	C9—C9^i^	1.522 (2)
C5—H5	0.95	C9—H9A	0.99
C6—O1	1.2317 (14)	C9—H9B	0.99
C6—N1	1.3383 (15)	O2—H2	0.950 (19)
N1—N3	1.4178 (14)		
			
C2—C1—C5	118.13 (10)	C2—N2—C3	118.21 (10)
C2—C1—C6	122.68 (10)	N1—N3—H3A	106.8 (8)
C5—C1—C6	119.16 (10)	N1—N3—H3B	105.7 (9)
N2—C2—C1	122.79 (10)	H3A—N3—H3B	105.8 (12)
N2—C2—H2A	118.6	O3—C7—O2	123.24 (11)
C1—C2—H2A	118.6	O3—C7—C8	124.36 (10)
N2—C3—C4	122.64 (11)	O2—C7—C8	112.40 (10)
N2—C3—H3	118.7	C7—C8—C9	115.55 (10)
C4—C3—H3	118.7	C7—C8—H8A	108.4
C5—C4—C3	119.13 (11)	C9—C8—H8A	108.4
C5—C4—H4	120.4	C7—C8—H8B	108.4
C3—C4—H4	120.4	C9—C8—H8B	108.4
C4—C5—C1	119.08 (11)	H8A—C8—H8B	107.5
C4—C5—H5	120.5	C8—C9—C9^i^	112.33 (12)
C1—C5—H5	120.5	C8—C9—H9A	109.1
O1—C6—N1	122.82 (11)	C9^i^—C9—H9A	109.1
O1—C6—C1	120.92 (10)	C8—C9—H9B	109.1
N1—C6—C1	116.25 (10)	C9^i^—C9—H9B	109.1
C6—N1—N3	122.12 (10)	H9A—C9—H9B	107.9
C6—N1—H1	120.3 (8)	C7—O2—H2	110.9 (10)
N3—N1—H1	116.6 (8)		
			
C5—C1—C2—N2	0.84 (16)	C5—C1—C6—N1	−143.68 (10)
C6—C1—C2—N2	178.88 (10)	O1—C6—N1—N3	−3.28 (17)
N2—C3—C4—C5	0.17 (17)	C1—C6—N1—N3	175.83 (10)
C3—C4—C5—C1	1.15 (17)	C1—C2—N2—C3	0.45 (15)
C2—C1—C5—C4	−1.61 (15)	C4—C3—N2—C2	−0.97 (15)
C6—C1—C5—C4	−179.73 (10)	O3—C7—C8—C9	−14.84 (18)
C2—C1—C6—O1	−142.57 (11)	O2—C7—C8—C9	165.28 (10)
C5—C1—C6—O1	35.46 (15)	C7—C8—C9—C9^i^	179.74 (12)
C2—C1—C6—N1	38.30 (15)		

Symmetry codes: (i) −*x*+1, −*y*+2, −*z*+2.

### Hydrogen-bond geometry (Å, °)

**Table:**

*D*—H···*A*	*D*—H	H···*A*	*D*···*A*	*D*—H···*A*
N1—H1···O3^ii^	0.877 (14)	2.174 (15)	3.0409 (14)	169.7 (11)
N3—H3A···O1^iii^	0.934 (14)	2.129 (14)	3.0349 (14)	163.0 (12)
N3—H3B···O1^iv^	0.888 (15)	2.258 (16)	3.1426 (14)	173.7 (13)
O2—H2···N2	0.950 (19)	1.671 (19)	2.6126 (13)	170.3 (16)
C2—H2A···O3	0.95	2.73	3.3838 (14)	126.

Symmetry codes: (ii) *x*, −*y*+3/2, *z*−1/2; (iii) −*x*, *y*+1/2, −*z*+1/2; (iv) −*x*, −*y*+1, −*z*.

## References

[bb1] Bernstein, J., Davies, R. E., Shimoni, L. & Chang, N.-L. (1995). *Angew. Chem. Int. Ed. Engl.* **34**, 1555–1573.

[bb2] Brandenburg, K. (1999). *DIAMOND* Crystal Impact GbR, Bonn, Germany.

[bb3] Farrugia, L. J. (1997). *J. Appl. Cryst.* **30**, 565.

[bb4] Farrugia, L. J. (1999). *J. Appl. Cryst.* **32**, 837–838.

[bb5] Friščić, T., Childs, S. L., Rizvi, S. A. A. & Jones, W. (2009). *CrystEngComm*, **11**, 418–426.

[bb6] Karki, S., Friščić, T. & Jones, W. (2009). *CrystEngComm*, **11**, 470–481.

[bb7] Lemmerer, A., Bernstein, J. & Kahlenberg, V. (2011). *CrystEngComm*, **13**, 55–59.

[bb8] Oxford Diffraction (2006). *CrysAlis PRO* Oxford Diffraction Ltd, Abingdon, England.

[bb9] Sheldrick, G. M. (2008). *Acta Cryst.* A**64**, 112–122. 10.1107/S0108767307043930 18156677

[bb10] Skovsgaard, S. & Bond, A. D. (2009). *CrystEngComm*, **11**, 444–453.

[bb11] Spek, A. L. (2009). *Acta Cryst.* D**65**, 148–155. 10.1107/S090744490804362X PMC2631630 19171970

